# Microfluidics-Based Analysis of Contact-dependent Bacterial Interactions

**DOI:** 10.21769/BioProtoc.2970

**Published:** 2018-08-20

**Authors:** Robert Cooper, Lev Tsimring, Jeff Hasty

**Affiliations:** 1 BioCircuits Institute, University of California, San Diego, La Jolla, CA, United States;; 2 San Diego Center For Systems Biology, University of California, San Diego, La Jolla, CA, United States;; 3 Division of Biological Science, University of California, Molecular Biology Section, La Jolla, CA, United States;; 4 Department of Bioengineering, University of California, San Diego, La Jolla, CA, United States

**Keywords:** Microfluidics, Horizontal gene transfer (HGT), Type VI secretion system (T6SS), Natural competence, Antibiotic resistance, Acinetobacter, Biofilm, Microbial ecology

## Abstract

Bacteria in nature live in complex communities with multiple cell types and spatially-dependent interactions. Studying cells in well-mixed environments such as shaking culture tubes or flasks cannot capture these spatial dynamics, but cells growing in full-fledged biofilms are difficult to observe in real time. We present here a protocol for observing time-resolved, multi-species interactions at single-cell resolution. The protocol involves growing bacterial cells in a near monolayer in a microfluidic device. As a demonstration, we describe in particular observing the dynamic interactions between *E. coli* and *Acinetobacter baylyi.* In this case, the protocol is capable of observing both contact-dependent lysis of *E. coli* by *A. baylyi* via the Type VI Secretion System (T6SS) and subsequent functional horizontal gene transfer (HGT) of genes from *E. coli* to *A. baylyi*.

## [Background]

Spatially-dependent interactions between different species of bacteria likely occur ubiquitously in nature, but they can be difficult to observe. One example is enhancement of horizontal gene transfer (HGT) by contact-dependent, *in situ* lysis of a prey cell, which serves as DNA donor, by a naturally competent, predatory, DNA recipient cell. This was only recently observed in Gram-negative bacteria, but it has already been seen in multiple species, and it is thought to be a relatively widespread phenomenon (Borgeaud *et al*., 2015; Cooper *et al*., 2017; Veening and Blokesch, 2017; Ringel *et al*., 2017). Killing-enhanced HGT cannot easily be observed at single-cell resolution in shaking culture tubes, both because single cells cannot be observed over time, and the well-mixed environment prevents spatial structure. These interactions occur in biofilms, but it is difficult to observe and track cells in their interior. Cells pressed between a glass slide and an agar pad are constrained to a two-dimensional spatial structure and can be observed during contact-dependent lysis (LeRoux *et al*., 2012; Basler *et al*., 2013). However, this method allows only a limited duration of observation before either nutrients are depleted, stopping cell growth, or growing colonies push up the agar and develop three-dimensional structure. Microfluidics is an ideal solution to these problems, as it continually provides fresh media while washing away excess, growing cells. The commonly used polydimethylsiloxane (PDMS) and glass substrates are rigid enough to maintain cells in an easily visualized monolayer, while still allowing complex dynamics that can approximate biofilm growth (Liu *et al*., 2015; Humphries *et al*., 2017). While we specifically describe contact-dependent killing and HGT between *A. baylyli* and *E. coli,* this method should also be generalizable to other species and spatially-structured interactions.

## Materials and Reagents

**Figure 1.**
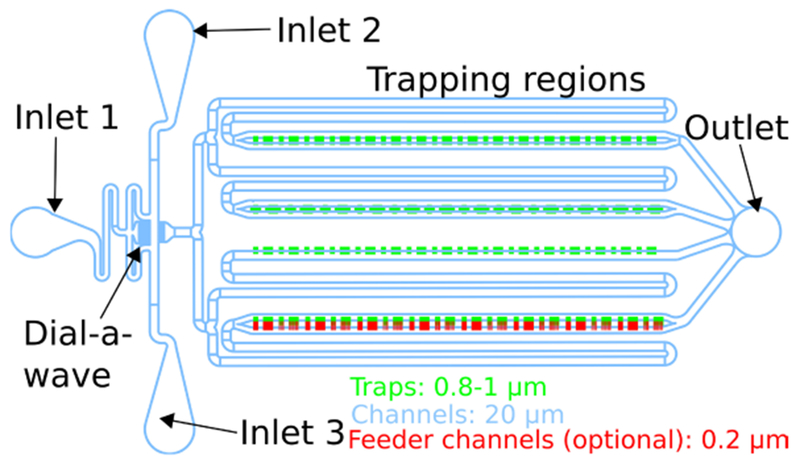
A microfluidic chip with four rows of traps and 3 media ports to allow dynamic media switching. Channels shown in blue are 20 μm tall, traps shown in green are 0.8–1 μm tall, and an optional layer of feeder channels shown in red are 0.2 μm tall. The three inlets allow precise concentrations of a dynamically varying chemical of choice, via the Dial-A-Wave mechanism. Note that the feeding channel layer and use of more than one inlet are optional.

**Figure 2.**
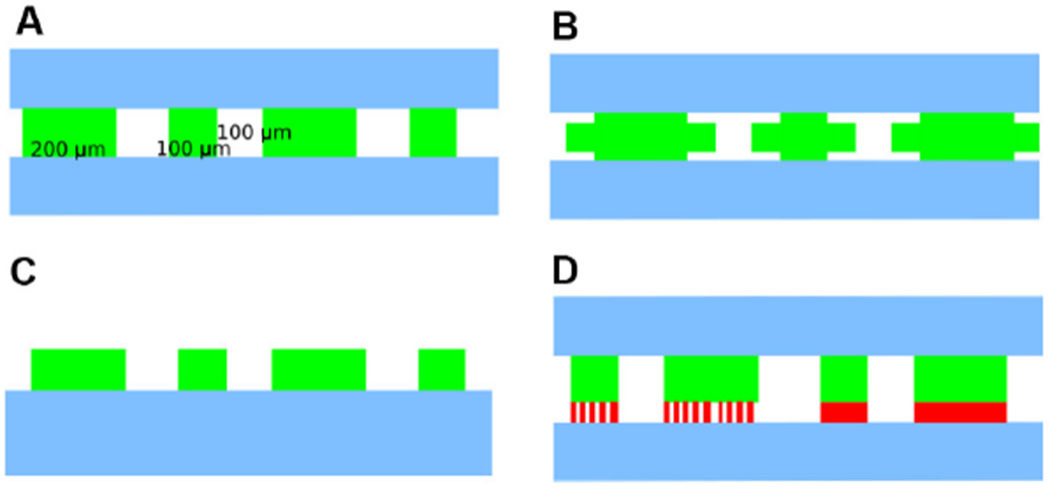
A closer view of the four rows of trapping regions. A. The top row contains 100 μm wide traps that alternate between 100 and 200 μm long, open to channels on two sides. B. The second row contains the same trap design with additional wings on the side that experience lower flow. C. The third row of traps is the same as the first, but open on only one side. D. The fourth row of traps is the same as the third, but the back is connected to another media channel by an optional third 0.2 μm tall layer to allow media exchange without allowing cells through. Note that this very low-height layer is technically difficult both to fabricate on a wafer (mold) and to prevent from collapsing on an assembled PDMS chip.

1. Glass coverslips, #1.5 (Fisher Scientific, catalog number: 12-530F)
2. Plastic weighing boat, hexagonal (Fisher Scientific, catalog number: 02-202B)
3. Razor blade (Fisher Scientific, catalog number: 12-640)
4. 0.5 mm biopsy punch (World Precision Instruments, catalog number: 504528)
5. Cutting mat (Harris, GE Healthcare, Whatman, catalog number: WB100020)
6. Clear removable tape (e.g., Scotch Magic Tape, catalog number: B0000DH8HQ)
7. Aluminum foil (*e.g*., Reynolds Recycled, catalog number: B0028LZ86A)
8. Squirt bottles containing 70% ethanol, water, methanol, and heptane, respectively (Fisher Scientific, catalog number: 03-409-34)
9. Parafilm (optional) (Bemis, catalog number: PM996)
10. 15 ml conical tubes (Genesee Scientific, catalog number: 21-103)
11. 1.5 ml microcentrifuge tubes (Fisher Scientific, catalog number: 05-408-129)
12. Microfluidic mold or chip
  Our layout is shown in Figure 1, and a mask design for photolithography is provided as supplementary information (see Co-culture chip.zip. Design files for a microfluidic chip for co-culturing different species of bacteria. The chip includes several different trap designs and geometries to facilitate experiment optimization). The specific design of a microfluidic chip is not critical, but some key considerations are as follows:
  - Trapping regions should be large enough to contain enough cells to make interesting dynamics likely, but they should also be small enough to allow sufficient diffusion of nutrients throughout the trap. We used several trap geometries with areas on the order of 10^4^ μm^2^.
  - Trap heights should maintain a cell monolayer. We used approximately 0.8-1 μm.
  - The fluid channels should avoid right angles and dead spaces, which contribute to clogging.
  Note: Our chip was based on the design developed in Danino et al. (2010), with parallel main media channels that have trapping chambers on their sides. We included multiple trap geometries, including some with one edge open to media channels, some with two edges open, some with additional, low-flow wings, and some with media feeder channels at the back, so we could test them all in parallel (Figure 2). The chip also includes a Dial-A-Wave design that allows precise control of the ratios of two different media sources. For single-media experiments as described here, it is sufficient to only use one inlet port, leaving the others un-punched. For further design considerations and details on media switching, see Ferry et al. (2011). If you have a pre-fabricated chip, you do not need items 1-16, and items 18-21 may need to be adjusted as appropriate for use with your chip.
13. BD 60 ml Luer-Lok syringes (BD, catalog number: 309053)
14. Tubing, 0.02” ID, 0.06” OD (Tygon, Murdock Industrial, catalog number: AAD04103)
15. Straight luer stubs, 23 ga × 0.50 in (Instech Laboratories, catalog number: LS23S)
16. Bent luer stubs, 23 ga × 0.50 in, bent 90° (OK International, Metcal, catalog number: 923050-90BTE)
17. *E. coli* carrying a plasmid that can transfer from *E. coli* to *A. baylyi*
  Note: We used pBAV1k-GFP (Addgene, catalog number: 26702), a plasmid with kanamycin resistance and a broad host range origin of replication that can propagate in both species (Bryksin and Matsumura, 2010). Another option would be to use a plasmid with the ColE1 origin of replication, which cannot propagate in A. baylyi, but to insert a GFP gene (and optionally the antibiotic resistance marker) between two flanking regions of A. baylyi genomic homology. This would encourage homologous recombination into the A. baylyi genome, which occurs at higher frequency than transfer of a self-replicating plasmid (Palmen et at, 1993). A good strain of E. coli for use in microfluidics is MG1655 (Coli Genetic Stock Center, catalog number: 6300), although other strains will work as well. Common cloning strains with mutations in recA, including dh5-alpha, are not ideal because they grow more slowly.
18. *A. baylyi* strain ADP1 (ATCC, catalog number: 33305), see also Notes
  Note: Either A. baylyi or E. coli or both should contain a fluorescent marker such as mCherry to visually distinguish between the two in movies. We inserted mCherry into a neutral region of the A. baylyi genome using a modified version of pp2.1-PT5-lacl-gusA-specR-pp2.2-1MBB (Murin et at, 2011; Addgene, catalog number: 30505) with the lacl-gusA insert replaced by mCherry.
19. Tween 20, aka, polysorbate 20 (Sigma-Aldrich, catalog number: P9416-50ML)
20. Dehydrated LB (Luria-Bertani) broth, Miller (BD, catalog number: 244610)
21. 70% ethanol (Fisher Scientific, catalog number: BP8201500).
22. Distilled water
23. Methanol (Fisher Scientific, catalog number: A412-500)
24. Heptane (Fisher Scientific, catalog number: H350-1)
25. Antibiotics as appropriate to maintain your cell strains
  - For *E. coli* carrying pBAV1k, use kanamycin at 50 μg/ml.
  - For *A. baylyi* with a marker inserted using a variant of pp2.1-PT5-lacl-gusA-specR-pp2.2-IMBB, use spectinomycin at 17 μg/ml.
26. Sylgard 184 silicone elastomer kit *(i.e*., polydimethylsiloxane or PDMS) including both base and curing agent (Ellsworth Adhesives, catalog number: 184 SIL ELAST KIT 0.5KG) (For Recipe 1)
  Manufacturer: Dow Corning, catalog number: 4019862.
27. PDMS (See Recipe 1)
28. LB + Tween 20 media (see Recipe 2)

## Equipment

1. Tweezers (*e.g*., Sports Medica, catalog number: B075VSFWSV)
2. Glass stirring rod for PDMS (*e.g*., United Scientific Supplies, catalog number: GSR008)
3. Vacuum desiccator for degassing PDMS (*e.g*., Thermo Fisher Scientific, Nalgene polypropylene desiccator with stopcock, catalog number: 5310-0250)
  Note: We attach this to an in-house vacuum line, but a vacuum pump would also work.
4. Oven set to 80 °C (*e.g*., Isotemp 500, Fisher Scientific, catalog number: 13246516GAQ)
5. UVO cleaner attached to an oxygen tank, used to bond chips to the cover glass (*e.g*., Jelight, model: 42)
6. Dissecting microscope for punching holes in the PDMS (*e.g*., Amscope, model: SM-4B)
7. Goose-neck illuminator for use with the microscope (*e.g*., Amscope, model: LED-6W)
8. Fume hood
9. Appropriate personal protective equipment
  Note: Appropriate personal protective equipment should be worn throughout, including a lab coat, nitrile gloves, and safety glasses.
10. Inverted, fluorescent microscope (We used Nikon TE and TI models)
  Note: An inverted, fluorescent microscope capable of automated imaging, including a CCD camera, a fluorescent light source, and appropriate filters.
11. Computer
  Note: A computer connected to the microscope and camera that is running software capable of controlling and receiving data from them, such as Micro-Manager (free and open source) or NIS-Elements, and ImageJ (free) to process image files.

## Software

1. ImageJ (https://imagej.nih.gov/ij/) or its packaged version FIJI (https://fiji.sc/)

## Procedure

**Figure 3.**
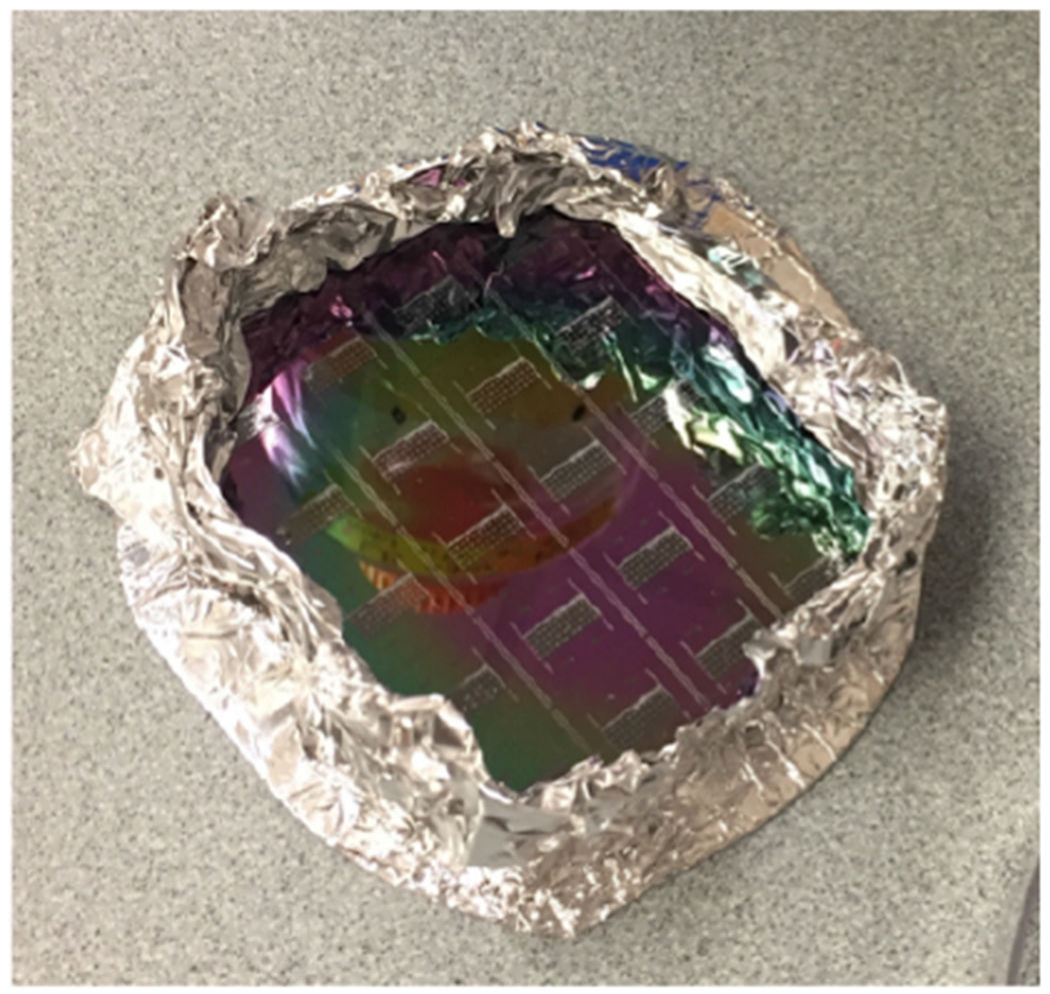
A wafer with molds of microfluidic chips ready for PDMS to be poured on

**Figure 4.**
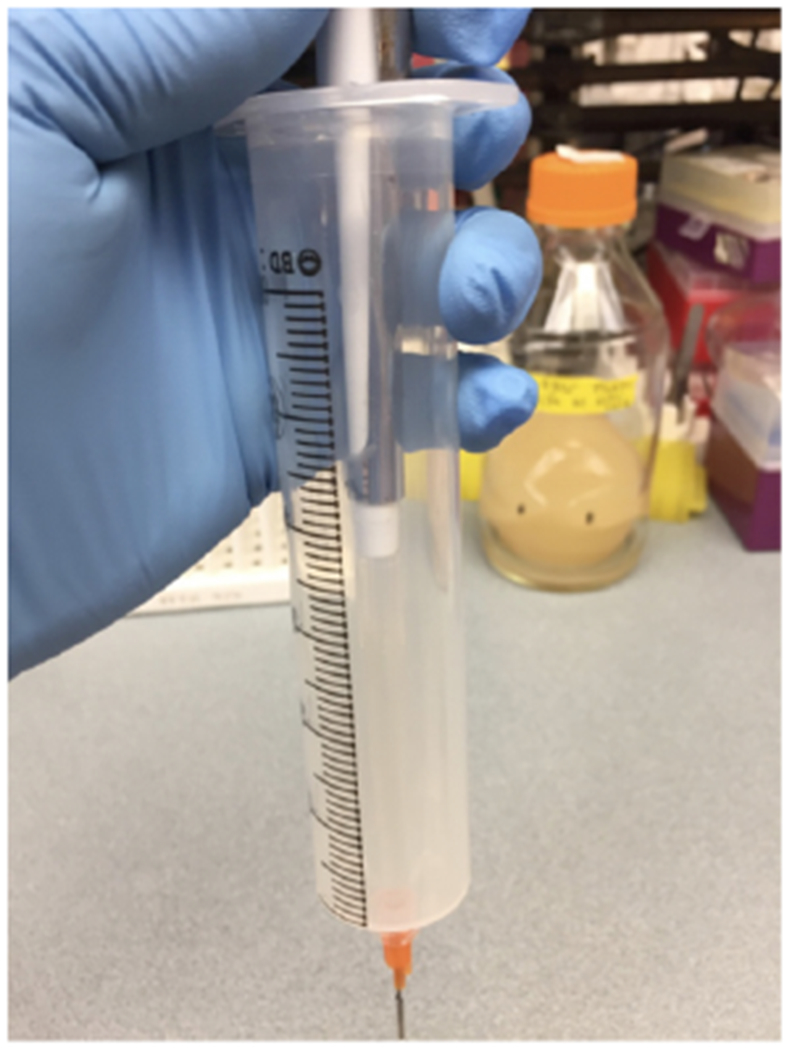
Prime the tubing by injecting media directly into the luer stub, through the bottom of the syringe

**Figure 5.**
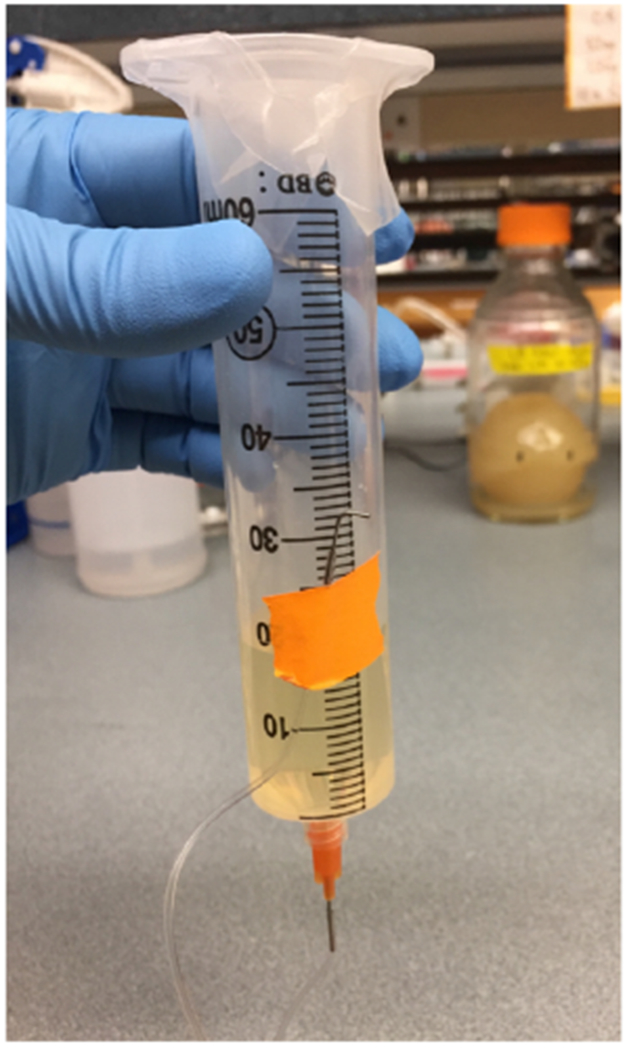
An example of a primed syringe

**Figure 6.**
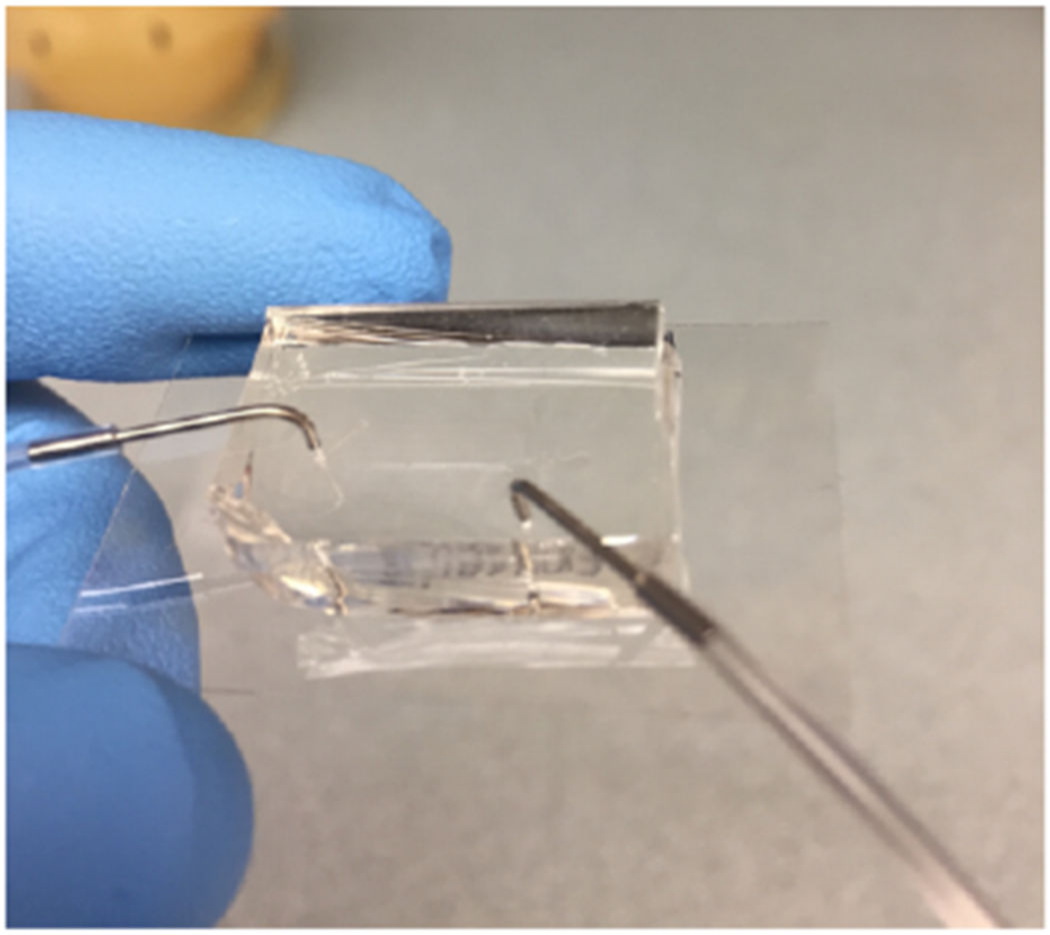
A loaded PDMS chip with 2 ports

**Figure 7.**
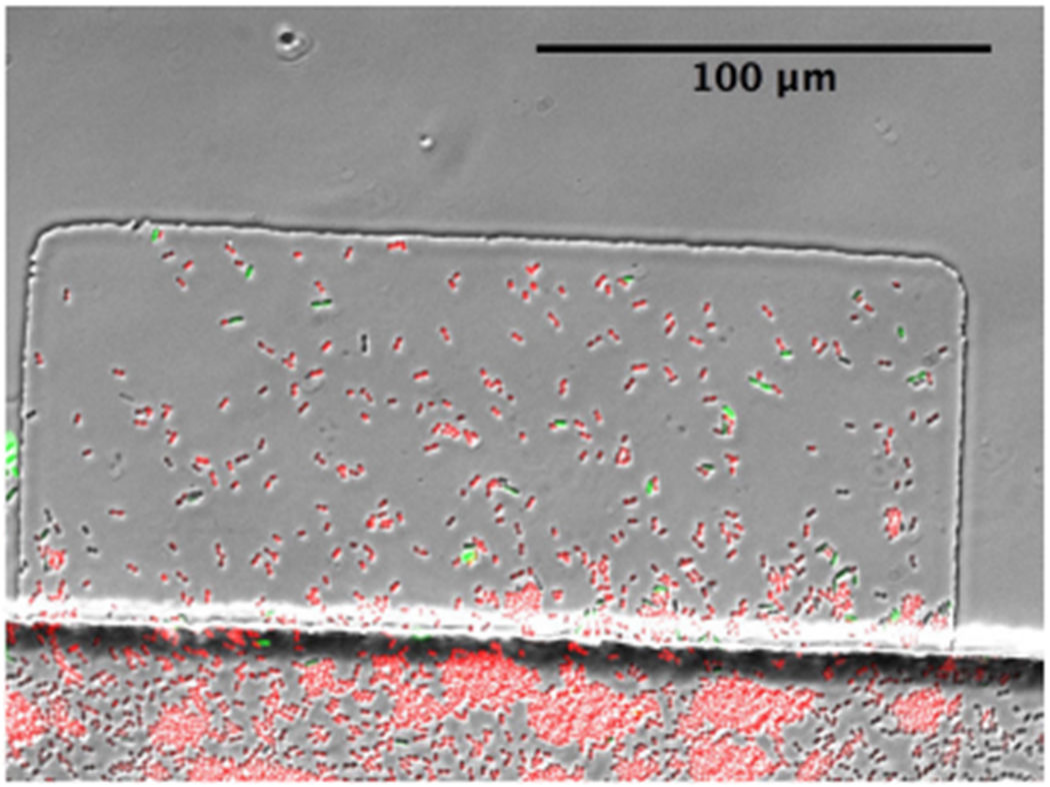
A microfluidic trap loaded with cells. The channel runs along the bottom of the image, *A. baylyi* cells are shown in red, and *E. coli* cells are shown in green.

- Fabricate a microfluidic chip
  Note: This section can be skipped if you have a pre-fabricated microfluidic chip.
  1. Prepare PDMS
    - Mix PDMS base and curing agent in a weigh boat at a ratio of 10:1; *i.e*., 36.4 g PDMS and 3.6 g curing agent. Mix thoroughly with a glass stirring rod.
    - Place the boat in the vacuum desiccator to degas the PDMS. When air bubbles are nearly overflowing the weigh boat, break the vacuum momentarily to pop and deflate the bubbles. Continue until there are no more bubbles.
  2. Prepare wafer
    Tear off a piece of aluminum foil and wrap it around the wafer containing your trap molds. Carefully press down, avoiding the mold features, to remove air bubbles (see Figure 3).
  3. Pour and cure PDMS
    Pour the degassed PDMS onto the wafer, and place it back into the vacuum desiccator to degas the PDMS as before. When the PDMS is thoroughly degassed, cure it in an 80 °C oven for an hour or overnight. Allow the cured PDMS to cool before proceeding, to avoid damaging your wafer.
  4. Cut and punch chips
    - Carefully peel the cured and cooled PDMS away from the wafer. Cut out individual chips with a razor blade on a cutting mat.
    - Turn the chips feature site up and place under the dissecting microscope. Carefully use the biopsy punch to remove a core all the way through the chip at each media port. Note that an experiment with no media switching only requires punching one inlet and one outlet.
  5. Clean and bond chips
    - Clean the chips sequentially with 70% ethanol and water, and dry them with a stream of nitrogen gas. Be sure to clean out the ports either with high-velocity gas or by injecting 70% ethanol or water through them using a syringe and luer stub adapter. Immediately place tape over the features of each chip after drying to prevent dust from settling on it.
    - In a fume hood, clean the coverslips sequentially with heptane, methanol, and distilled water before drying under a stream of nitrogen gas. Immediately place tape over each coverslip after drying to prevent dust from settling on them.
    - Open the oxygen line to the UVO cleaner. Place the chips (feature side up) and coverslips inside the UVO cleaner, peel off the tape, and activate the surface for 3 min. Re-close the valve of the oxygen tank.
    - Quickly and carefully place each chip feature side down onto a coverslip, and then bake at 80 °C for an hour or overnight to bond them.
      Note: If your chips have low height features (like the 1 μm traps on our chips), they can collapse if you press down on the chip. Placing the coverslip on top of the chip instead of the other way around can help protect low features.
- Prepare chip and media
  1. Note that our design includes an optional media switcher requiring 3 inlet ports. For an experiment with no media switches, it is sufficient to use only one inlet and one outlet, leaving the remaining inlets un-punched. For details on media switching, see Ferry *et al.* (2011). For each port, remove the metal part of a bent luer stub adapter. This can be done by soaking the adapter in acetone to dissolve the adhesive, and then pulling out the metal piece with tweezers. Discard the plastic and keep the metal piece.
  2. For each port, attach a straight luer stub adapter to a 50 ml syringe. Attach a length of tubing to the adapter, and insert the metal piece from a bent luer stub adapter into the other end of the tubing.
  3. Prime the tubing of all but one syringe by injecting 1 ml of LB with Tween 20 (see Recipes) straight down into the bottom of the syringe (see Figure 4). Press the pipet tip against the very bottom of the syringe, and then maintain steady pressure on the plunger until you see LB begin to come out the other end of the tubing. Carefully add another 10 ml of LB into the syringe. Allow fluid to flow through the tubing until you are sure there are no more bubbles in the line, and then tape the end of the tubing to the syringe just above the liquid level to stop the flow. Cover the top of the syringe with tape or parafilm to prevent contamination (see also Figure 5). Note that chilled media will release dissolved gases as it warms, so to avoid bubbles, allow any chilled media to warm to at least room temperature before loading your syringes. See also Video 1, in which the syringe is loaded with food coloring to aid visualization.
- Prepare cells
  1. Meanwhile, grow 10-15 ml of each strain separately in LB with appropriate antibiotics to mid-exponential phase. For *E. coli* carrying pBAV1k, use kanamycin at 50 μg/ml.
  2. Harvest each strain by transferring to 15 ml conical tubes and centrifuging for 5 min at 2,000 × *g.* Room temperature is fine for centrifugation steps.
  3. Resuspend each strain in 1 ml fresh LB, transfer to a 1.5 ml microcentrifuge tube, and harvest again for 3 min at 10,000 × *g.* This washing step is to remove any residual antibiotics.
  4. Resuspend the two strains at high density – use about 1-3 volumes of LB with Tween 20 for each volume of cell pellet.
  5. Mix the two strains at 3 volumes of *E. coli* for each volume of *A. baylyi.* The ratio can be adjusted in subsequent attempts, but *E. coli* MG1655 are less adhesive than *A. baylyi* and thus are more likely to be washed out of the traps during and immediately after loading. If using another *E. coli* strain that is more adhesive, a more equal ratio of the two species may work better. Note also that species ratio can affect the frequency of interactions such as horizontal gene transfer (Cooper *et al*., 2017).
  6. Load the cell mixture into a prepared syringe and tubing in the same way as for the media syringes above.
- Load the chip
  1. For each media port, lower the luer stub below the fluid level in the syringe, hold it until fluid begins flowing out from the end, and insert it into the appropriate port in the chip, as in Figure 6. Do the same for the cell mixture in the waste port. Note that the loading speed can be increased by placing the chip in the vacuum desiccator for at least 20 min before loading.
  2. Place the chip onto the microscope stage, fix it in place, and raise the syringes a few feet above the chip. They can be taped to the wall, or for a more advanced setup see Ferry *et al.* (2011). Watch under the microscope as the channels fill with media. You want the fresh media and the cells to meet about midway into the trapping region. The extra buffer space is to prevent contaminating the media channel with cells in the next step. Adjust the syringe heights to ensure this. When the media does meet the cells, adjust the syringe heights so there is a slow forward flow from media to waste.
  3. If cells have not loaded well into the traps, flick the waste media line (loaded with cells) with your fingers. Try gently at first, and then harder as needed (see also Video 2). The pressure waves should force cells into the traps, but be careful not to force cells into the upstream media channels.
  4. Once sufficient cells of each species are loaded into the traps (see Figure 7 for a representative example of a loaded trap), immediately adjust the syringe heights to obtain a forward flow from media to waste. The flow velocity must be fast enough to avoid clogging, but not so fast as to wash the less sticky *E. coli* out of the traps. The flow rate can be adjusted up as the cells grow and fill the traps. Both syringes should be above the chip.
- Record data and babysit the experiment
  1. Set your microscope to record one set of images every 3-5 min. You will need to determine the appropriate exposure power and duration to observe cellular fluorescence on your equipment. In general, use the lowest possible exposure that will give reliable data, to avoid damaging the cells. A longer exposure at lower intensity is preferable to a short exposure at high intensity. Imaging multiple stage positions using a motorized stage is ideal, because it increases the likelihood you will capture usable data.
  2. Keep an eye on the experiment to be sure that cells are growing and the channels do not clog. If cells are washing out of the traps, reduce the flow rate by raising the waste syringe or lowering the fresh media syringe. If channels are beginning to clog, or if media is not flowing forward, increase the flow rate by adjusting the syringe heights in the opposite direction.
  3. If channels begin clogging, this generally portends the beginning of the end. Sometimes, the experiment can be given a temporary reprieve by flicking the tubing connecting the syringes to the chip, as in Video 2. However, this can also rearrange the cells within the traps, which can compromise time course data. See also Notes.

## Data analysis

Captured still images can be converted into movies using the free program ImageJ, which also comes in a package recursively called FIJI.

1. Import a folder of images with the command *File > Import > Image Sequence.* Be sure to select ‘Sort names numerically’. Using a virtual stack (opening with the computer’s virtual memory rather than loading into RAM) will load faster and can be useful for large folders on computers with limited RAM, but annotations often do not work on virtual stacks.
2. Convert to a hyperstack using the *Images > Stacks > Stack to Hyperstack* command. Specify the appropriate number of positions, channels, time points, and Z-slices. Select display as composite.
3. Adjust the color and contrast of each channel using the tools available at *Image > Color > Channels Tool* and *Image > Adjust > Brightness And Contrast.*
4. Optional: If you want to add any annotations, such as time stamps or scale bars, you must convert the multichannel hyperstack to an RGB stack using *Image > Type > RGB Color.* Be sure to adjust the contrast first, because it will be fixed once the stack has been converted to RGB.
5. Optional: Add any desired annotations. To add time stamps, use *Image > Stacks > Label.* To add a scale bar, use *Analyze > Set Scale* followed by *Analyze > Tools > Scale Bar.*
6. Optional: Crop all frames in the movie by selecting the rectangle tool and then *Image > Crop.* Select only a portion of the time series using *Image > Stacks > Tools > Make Substack.*
7. Save your processed movie as a TIFF stack at full quality and/or as a compressed movie using the options in *File > Save* As. You may need to download a plugin to save as a .avi or .mov file from https://imagej.nih.gov/ij/plugins/. Saving as an animated GIF is another option that is easily shared on social media.
8. Representative results can be seen in the attached movies for Cooper *et al*., 2017.

## Notes

In our hands, *A. baylyi* was difficult to work with in microfluidics, because it adhered to both the PDMS and glass coverslips of our chips. We attempted several strategies to reduce cellular adhesion, including adding high levels of Tween 20, adding DNase (Das *et al*., 2010), adding PEG to the PDMS before curing, and deleting the thin pilus gene *acuA* (Gohl *et al*., 2006), but none helped significantly.

Interestingly, some of our experiments began well, but then there appeared to be a change in *A. baylyi* that caused them to become more adhesive to both the surfaces and each other. In a microfluidic device where non-adhesive cells are washed away, the adhesive phenotype is constantly selected for and rapidly dominates once it emerges. The adhesive phenotype may be related in part to recently described genomic instability caused by mobile genetic elements that cause genomic insertions and deletions at relatively high frequencies (Renda *et al*., 2015). When this instability disrupts production of bioemulsifier, the cells begin to aggregate. A strain of *A. baylyi* that lacks all insertion elements (Suárez *et al*., 2017) may work better in microfluidics, but we have not tested it. An alternative explanation may be a developmental switch between bacillar and coccoid phenotypes, with the coccoid phenotype being related to nutrient stress and adhesion (James *et al*., 1995).

Regardless, while cellular adhesion limited the duration of our experiments, we were able to run them for long enough to reproducibly observe T6SS-dependent killing and subsequent HGT before the channels fully clogged (Cooper *et al*., 2017).

## Recipes

1. PDMS
  36.4 g silicone elastomer
  3.6 g curing agent
  Weigh together in a plastic weigh boat and stir thoroughly with a glass stir bar immediately before proceeding to degas and pour onto the wafer
2. LB + Tween 20
  25 g LB powder
  Distilled water to 1 L
  Tween 20
  Dissolve the LB powder and Tween 20 into distilled water, then filter sterilize. Alternatively, add Tween 20 into pre-made LB liquid and filter sterilize

## Supplementary Material

Co-culture chip.zip

## Figures and Tables

**Video 1.**
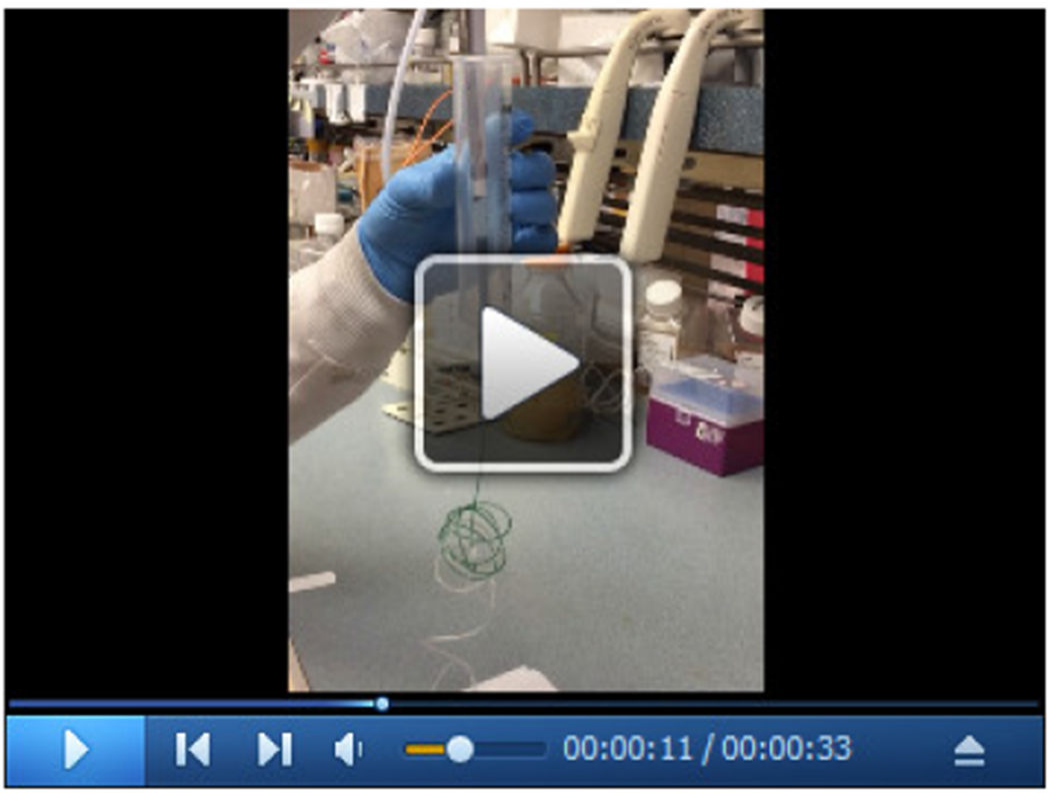
Loading a syringe. To aid visualization, this syringe was loaded with water dyed green with food coloring.

**Video 2.**
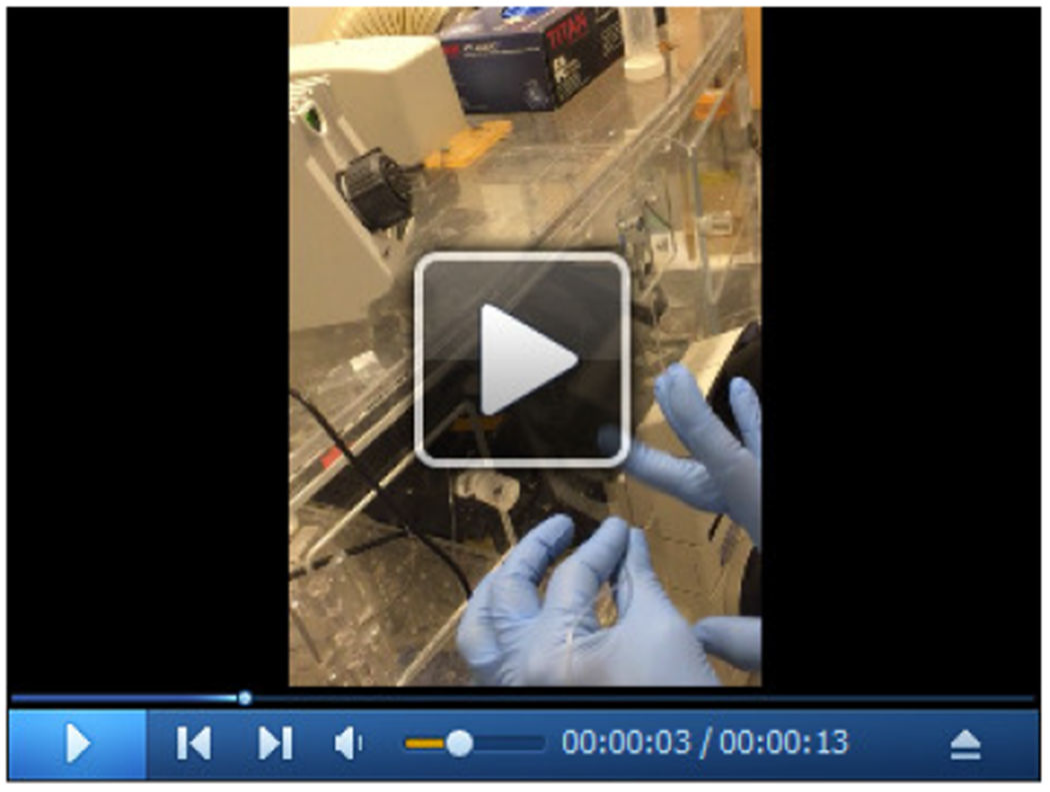
Flicking the microfluidics lines. A demonstration of how to flick the media lines, which helps initial loading of cells and can temporarily disrupt clogs.
