# Supplementary figures and images for: Microfluidics-Based Analysis of Contact-dependent Bacterial Interactions
Source: Bio Protoc. Author manuscript; Available in PMC 2018 Oct 24. (PMC6200413; doi:10.21769/BioProtoc.2970)

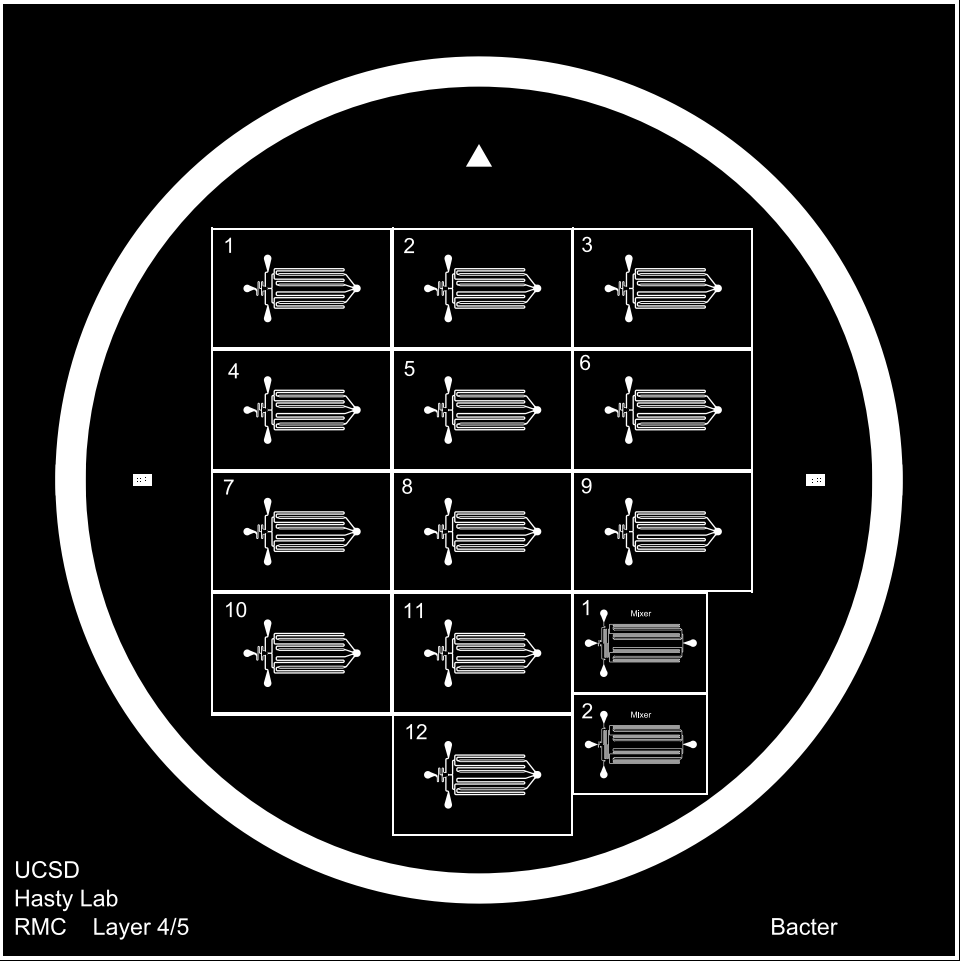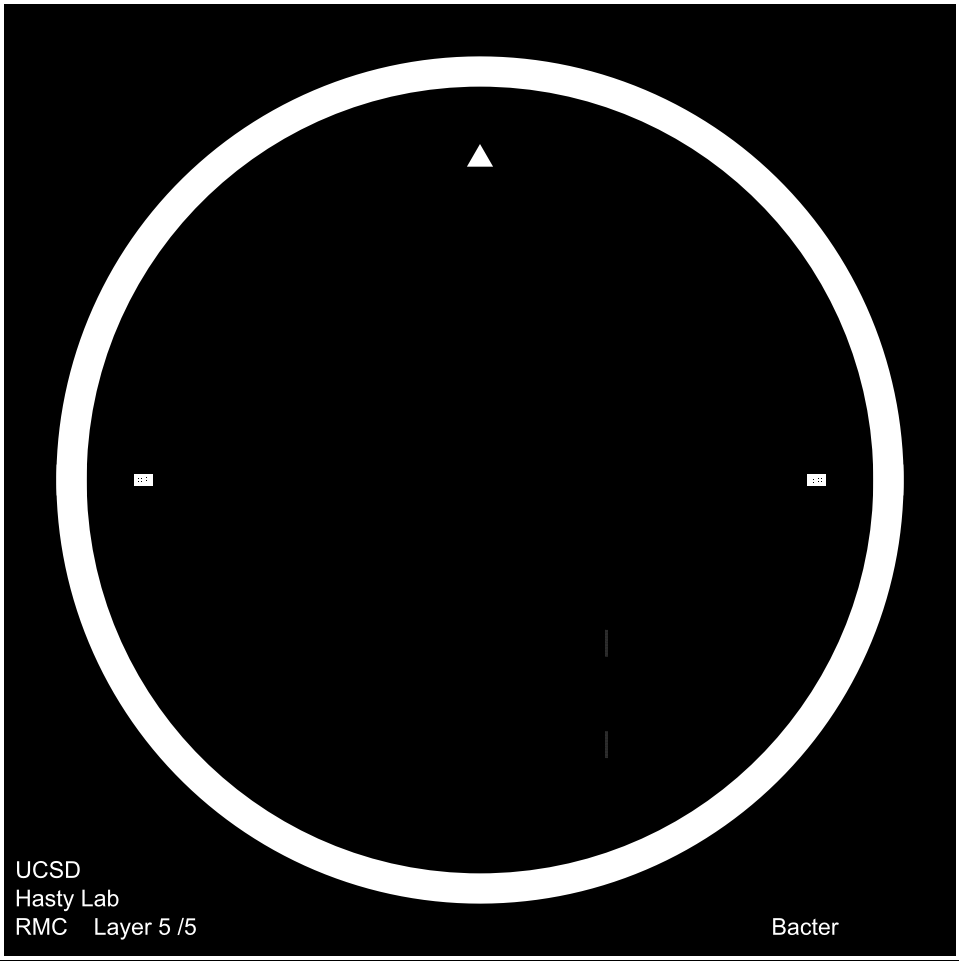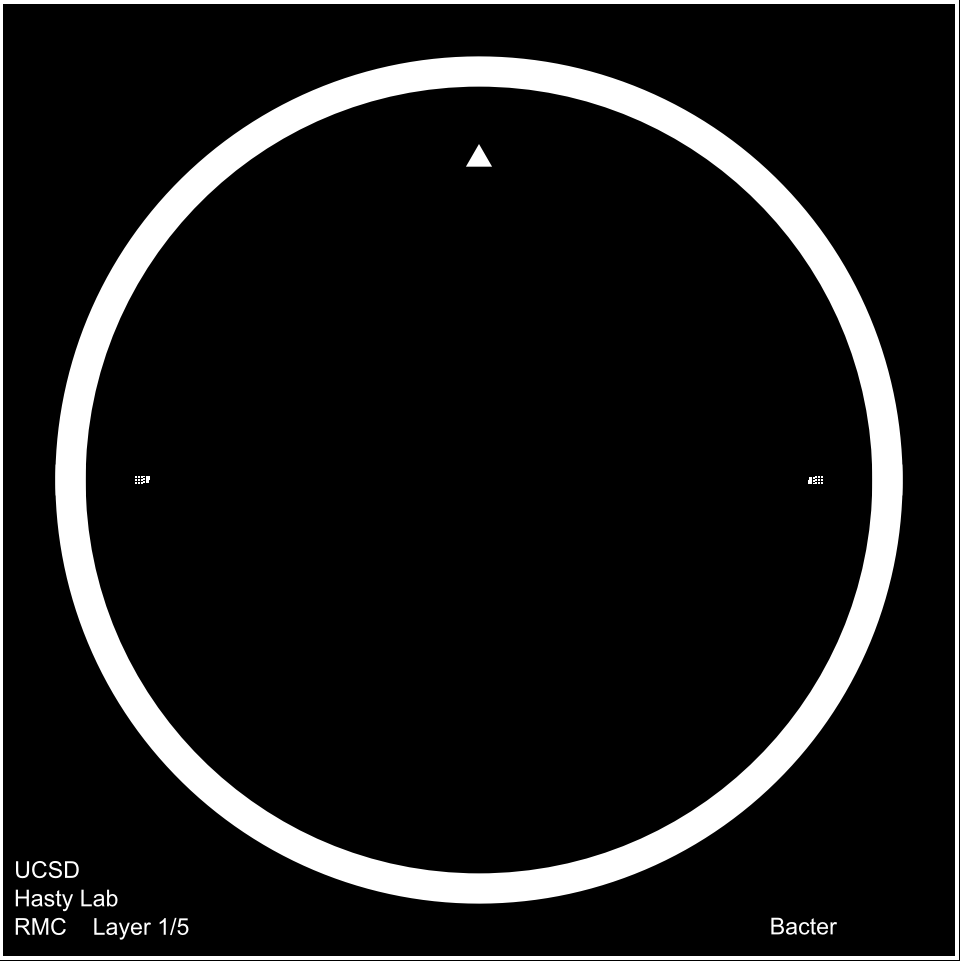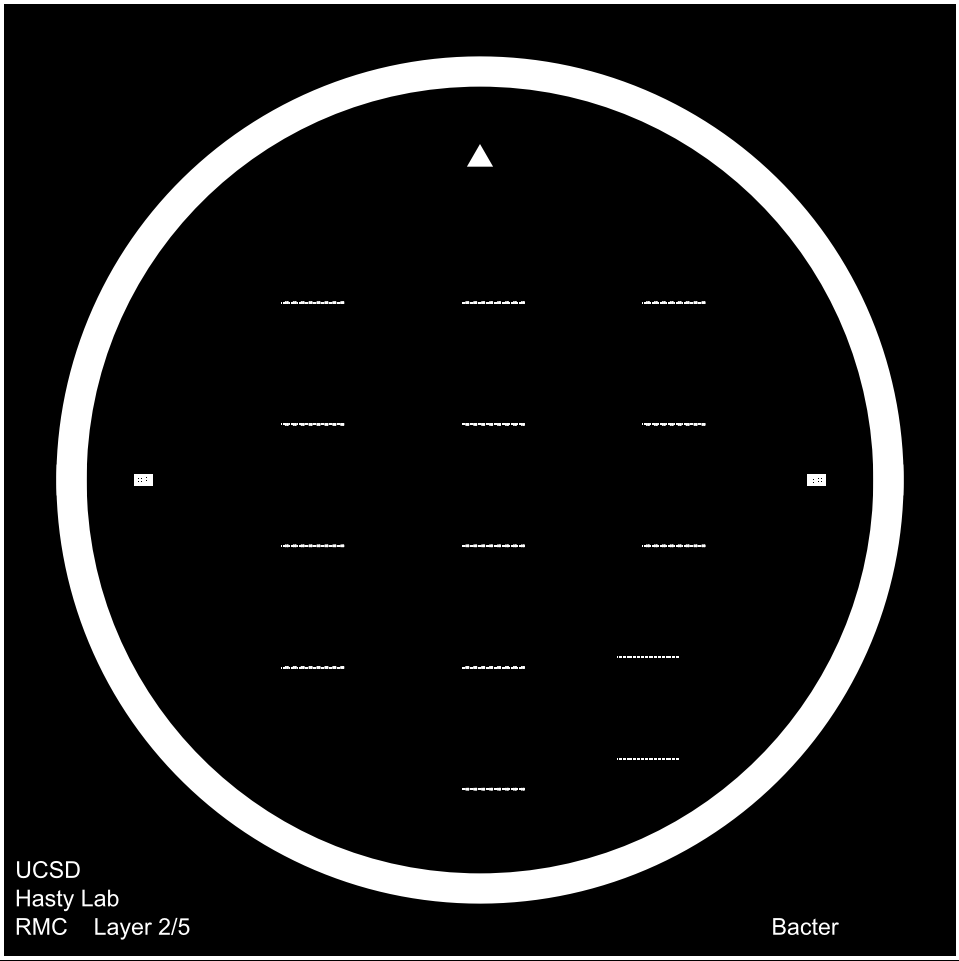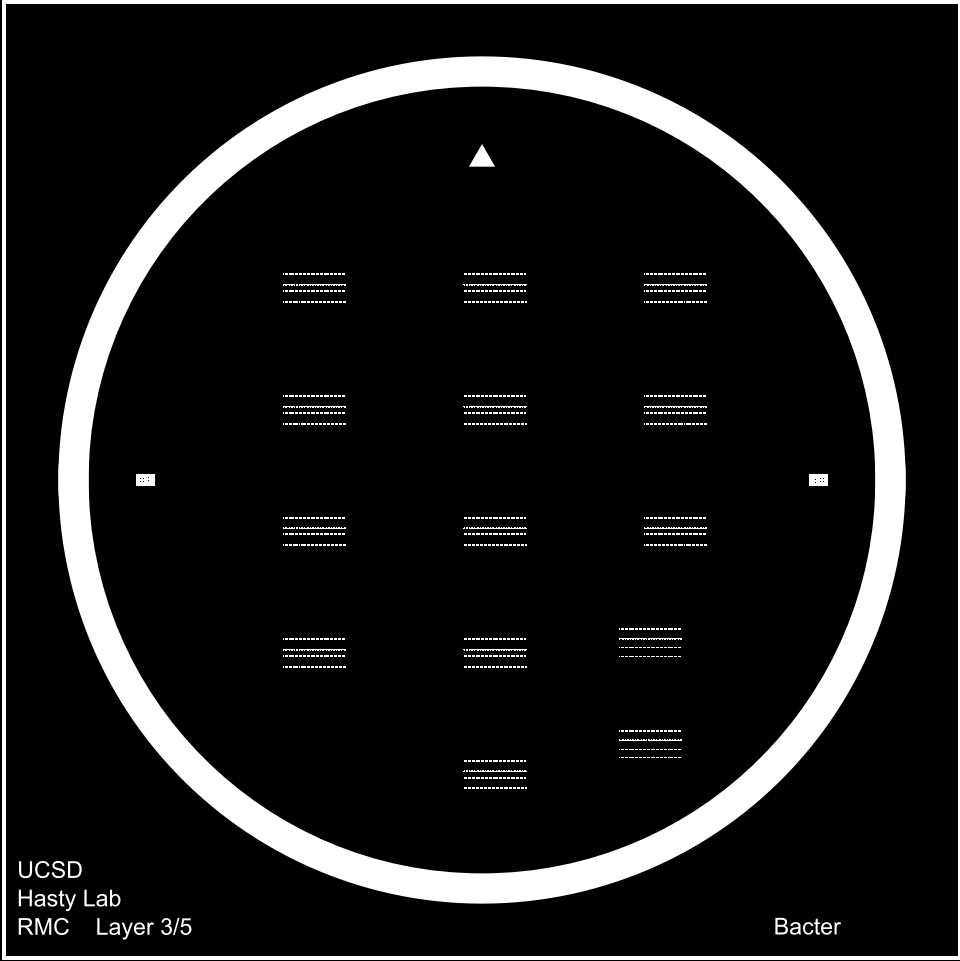

Supplement: Co-culture chip.zip [file NIHMS987013-supplement-Co-culture_chip_zip.zip › adp1-chip.pdf]
